# Widely Targeted Metabolomics Analysis Reveals Developmental Shifts in Antioxidants and Functional Peptides in *Akebia trifoliata*

**DOI:** 10.3390/antiox14091039

**Published:** 2025-08-24

**Authors:** Tianjiao Jia, Mian Faisal Nazir, Edgar Manuel Bovio-Zenteno, Longyu Dai, Jie Xu, Yafang Zhao, Shuaiyu Zou

**Affiliations:** 1 Lushan Botanical Garden, Jiangxi Province and Chinese Academy of Sciences, Jiujiang 332900, China; jiatj@lsbg.cn (T.J.); mfn121@hotmail.com (M.F.N.); boviozentenoedgar@gmail.com (E.M.B.-Z.); dlongyu1997@163.com (L.D.); xj13707060350@163.com (J.X.); fang10302021@163.com (Y.Z.); 2 Instituto de Biotecnología y Ecología Aplicada (INBIOTECA), Universidad Veracruzana, Av. Culturas Veracruzanas 101, Col. E. Zapata, Xalapa 91090, Veracruz, Mexico

**Keywords:** *Akebia trifoliata*, widely targeted metabolomics, secondary metabolites, bioactive compounds, oligopeptides

## Abstract

*Akebia trifoliata* is an emerging fruit crop in China, valued for its medicinal and nutritional properties. To elucidate the developmental dynamics of its bioactive compounds, we performed widely targeted metabolomics using Ultra Performance Liquid Chromatography–Tandem Mass Spectrometry (UPLC-MS/MS) across four fruit developmental stages (S1–S4). A total of 1595 metabolites were identified, of which 988 were differentially accumulated and categorized into three distinct accumulation patterns. Flavonoids and phenolic acids exhibited a marked decline during fruit maturation, corresponding with decreasing antioxidant and α-glucosidase inhibitory activities. Conversely, functional oligopeptides and specific terpenoids accumulated significantly at later stages. K-means clustering revealed dynamic shifts in metabolic profiles, and 23 functional oligopeptides with antioxidative, antidiabetic, and ACE-inhibitory activities (angiotensin-converting enzyme, ACE) were predicted. KEGG enrichment highlighted stage-specific pathway transitions from flavonoid biosynthesis during early development to sugar metabolism at ripening. Correlation analysis identified key flavonoids, phenolic acids, and amino acid derivatives associated with antioxidant capacity and α-glucosidase inhibition. This study provides comprehensive metabolomic landscape of *A. trifoliata* fruit development and offers valuable insights for its functional exploitation in food and medicinal applications.

## 1. Introduction

*Akebia trifoliata*, belonging to the *Akebia* genus of Lardizabalaceae and commonly known as “Bayuezha” in China, is a deciduous woody liana that is mainly distributed in East Asia, especially in China, Korea, and Japan [1]. Its ripe fruit has a sweet and juicy pulp containing multiple saccharides, crude proteins, amino acids, minerals, and vitamins [2,3]. The nearly ripe, dry fruit of *A. trifoliata* is an important traditional Chinese medicine and is described as Akebiae Fructus in the pharmacopoeia of China. The earliest recorded use of *A. trifoliata* as a traditional Chinese medicine was in “Shen Nong’s Herbal Classic” (around 100 BC), where it was mainly used for soothing the liver, promoting blood circulation, regulating Qi and relieving pain. Studies of the biological activity of *A. trifoliata* have confirmed its diuretic, neuroprotective, analgesic, anti-inflammatory, anti-obesity, anti-aging, antibacterial, antioxidative and anticancer activities [4]. Given its diverse pharmacological properties and increasing consumer demand, *A. trifoliata* has recently gained recognition as a promising candidate for both medicinal applications and functional food development, leading to its gradual introduction into commercial cultivation [5].

According to the literature, the main chemical constituents of *A. trifoliata* are triterpenoids, triterpenoid saponins, phenylethanoid glycosides, chlorogenic acids, and coumarins [6]. Terpenoids and triterpenoid saponins are the main chemical components and the material basis of pharmacological action of *A. trifoliata*. Several studies have performed qualitative and quantitative analyses the triterpenes in *A. trifoliata* using HPLC or LC-MS [7]. Most of these studies have concentrated on identifying and characterizing individual compounds, whereas investigations into the temporal variation in secondary metabolites and bioactive constituents during the fruit’s developmental stages remain limited.

Secondary metabolites play a vital role in the therapeutic efficacy of medicinal plants and are widely regarded as key markers for assessing the quality of medicinal raw materials [8]. However, the biosynthesis and accumulation of secondary metabolites are strongly affected by developmental stages and environmental factors [8]. Indeed, the developmental stages significantly influence the content and composition of components in most of medicinal plants. Pholphana et al. [9] reported that the main active ingredient in *Andrographis paniculata* is the diterpenoid 14-deoxyandrographolide (AP6), which is found at the highest level in the leaves at the transfer stage, an effect of the growth stages. The essential oil yields in *Citrus medica* L. var. *sarcodactylis* accumulate rapidly during the maturation process, and the content of carene, α-thujone, β-pinene, and γ-terpinene vary significantly during maturation stages [10]. Likewise, Xu et al. [11] reported that the concentration of major flavonoids in *Scutellaria baicalensis* roots rose substantially in the period leading up to full bloom. Farmers believe that the *A. trifoliata* fruits harvested in July–August have higher efficacy and maximize the use of the fruit. However, there is no concrete proof that *A. trifoliata* has the highest level of active components in this particular harvest period.

Metabolomics enables the large-scale analysis of metabolites, providing simultaneous qualitative and quantitative insights into the composition of metabolites in given biological samples, thereby revealing the overall dynamic changes in plant metabolism at different growth stages [12]. In recent years, UPLC-MS/MS-based widely targeted metabolomics has emerged as a powerful and dependable technique for comprehensive profiling and quantification of diverse plant metabolites with high precision and throughput [12,13]. This approach has been widely used in fruit metabolite analysis, such as kiwifruit, cherry, peach, and blueberry [14,15,16,17].

Although *A. trifoliata* has been widely recognized as a functional fruit with both medicinal and edible qualities [2,18,19,20], there are still few studies on the metabolite changes during the *A. trifoliata* maturing process, which impedes the breeding improvement and utilization of *A. trifoliata*. Thus, this study aimed to comprehensively characterize the dynamic shifts in chemical composition and antioxidant potential of *A. trifoliata* fruits across four distinct developmental stages. Using widely targeted metabolomics based on UPLC-MS/MS, we systematically profiled the secondary metabolites and identified differentially accumulated compounds throughout fruit maturation. The results offer both qualitative and quantitative insights into metabolic transitions during fruit development, providing a valuable foundation for optimizing the functional utilization and harvesting strategies of *A. trifoliata* based on its bioactive compound composition.

## 2. Materials and Methods

### 2.1. Plant Material

The present study was conducted using *Akebia trifoliata* (ssp. *australis*) plants cultivated in Jiujiang City, Jiangxi Province, China (29°38′ N, 115°59′ E). The vines, all five years old and grown under uniform management conditions with natural pollination, served as the plant material. Healthy fruits—free from pests, mechanical damage, and visible disease—were randomly collected in the germplasm resource at four developmental stages: S1 (60 days after full bloom, DAFB), S2 (90 DAFB), S3 (120 DAFB), S4 (150 DAFB), corresponding to fruit ripening in October. For each developmental stage, fifteen fruits (five per tree) were harvested and pooled into three biological replicates for downstream analysis. The whole fruits were cut into pieces, then rapidly frozen with liquid nitrogen, and subsequently stored in a −80 °C refrigerator for subsequent experiments.

### 2.2. Measurement of Fruit Morphological Traits

Immediately after harvest, key physical attributes of the fruits were measured. These included single fruit weight (SFW), fruit length (FL), fruit diameter (FD), and dry matter content (DM). SFW was recorded using a precision electronic balance, while FL and FD were determined with a digital caliper. The DM content was calculated by oven-drying the fruit samples and applying the standard drying–weighing procedure as described by [21]. Briefly, an electronic balance was used to weigh thin slices of fresh fruit, which were subsequently dried in an oven at 70 °C until their weights remained constant. The percentage of dry weight to fresh weight was used to calculate the dry matter content of *A. trifoliata*.

### 2.3. Sample Processing and Extraction

Fruit tissues were initially freeze-dried using a Scientz-100F vacuum lyophilizer (Scientz, Ningbo, China; condensation trap temperature: −60 °C; sample compartment temperature: −30 °C; pressure: 50 Pa; freeze-drying time 48 h) and then finely pulverized with a Retsch MM 400 mixer mill equipped with zirconia beads (particle size: 10 mm), the grinding jar and zirconia beads pre-cooled in liquid nitrogen, operating at 30 Hz for 90 s. A 50 mg aliquot of the powdered sample was accurately weighed using an electronic balance (XPR105/AC, Mettler Toledo, Switzerland) and extracted in 1.2 mL of 70% methanol (*v*/*v*) at 4 °C. The mixture was vortexed 30 s per 30 min, repeated 6 times. The extract was then centrifuged at 12,000 rpm, 4 °C for 3 min, and the resulting supernatant was passed through a 0.22 μm pore-size membrane filter (SCAA-104, ANPEL, Shanghai, China) before analysis by UPLC-MS/MS.

### 2.4. Quantification of the Total Phenolics and Flavonoids Content

The total phenolic content (TPC) was determined using the Folin–Ciocalteu colorimetric method, with gallic acid employed as the reference standard [3,21]. Approximately 0.5 g of freeze-dried sample powder was extracted in 75% methanol (*v*/*v*), using ultrasound-assisted extraction for 30 min (300 W, 50 KHZ). One milliliter of the resulting extract was mixed with an equal volume of Folin–Ciocalteu reagent and left to react for 5 min. This was followed by the addition of 8.0 mL of sodium carbonate solution (75 mg/mL), and the mixture was incubated in the dark for 1 h at 25 °C. Absorbance was measured at 765 nm using a spectrophotometer (BioSpEctrometer^®^ basic, Eppendorf, Germany). TPC was calculated using a standard curve for gallic acid (y = 0.004x + 0.0832, R^2^ = 0.9923) and expressed as mg gallic acid equivalents (GAE) per gram of dry weight. All assays were carried out in triplicate.

Total flavonoid content (TFC) was determined based on the aluminum chloride colorimetric assay, as described by [3]. Briefly, 0.5 g of sample was extracted with 80% ethanol (*v*/*v*), followed by centrifugation at 10,000× *g* for 20 min at 4 °C. From the clear supernatant, 0.5 mL was mixed with 0.1 mL of 50 g/L sodium nitrite. After a 5 min reaction, 0.1 mL of 10% aluminum chloride was added. Following an additional 6 min, 1.0 mL of 1 mM sodium hydroxide was added to complete the reaction. The mixture was kept at room temperature (25 °C) for 30 min, and absorbance was recorded at 510 nm using a spectrophotometer (BioSpEctrometer^®^ basic, Eppendorf, Germany). Flavonoid content was quantified using a rutin calibration curve and expressed as mg rutin equivalents (RE) per gram of dry weight.

### 2.5. Determination of Antioxidant Activities

Antioxidant extracts were prepared following the protocol of Geng et al. [22] with slight modification. Briefly, 0.5 g of powdered sample was extracted with 50 mL of 75% methanol using an ultrasonic bath (300 W, 50 KHZ) at 25 °C for 30 min. The mixture was then centrifuged at 10,000× *g* for 20 min at 4 °C. The resulting supernatant was used for evaluating antioxidant and α-glucosidase inhibitory activities.

Three standard assays, DPPH (2, 2-diphenyl-1-picrylhydrazyl), ABTS (2, 2-azinobis (3-ethylbenzthiazoline-6-sulfonic acid)), and FRAP (ferric reducing antioxidant power), were employed to assess the antioxidant capacity of the extracts.

For the DPPH and ABTS assays, the DPPH and ABTS working solutions were prepared following the protocol of Jia et al. [23]. Initially, 0.5 mL of the extract was mixed with 3.5 mL of DPPH or ABTS working solution. After incubation in the dark at room temperature for 30 min, absorbance was recorded at 517 nm (DPPH) and 734 nm (ABTS) using a spectrophotometer (BioSpEctrometer^®^ basic, Eppendorf, Germany). The radical scavenging activity (%) was calculated using the following formula:

Radical scavenging rate (%) = [(A_control_ − A_sample_)/A_control_] × 100%.

where A_control_ is the absorbance of the mixed solution containing 75% methanol and DPPH/ABTS solution in anhydrous ethanol, and A_sample_ is the absorbance of the mixed solution containing diluted extracts and anhydrous ethanol.

The ferric reducing antioxidant power (FRAP) assay was conducted following the procedure outlined by [24]. The FRAP working solution was freshly prepared by combining 300 mM acetate buffer (pH 3.6), 10 mM TPTZ (2,4,6-tripyridyl-s-triazine), and 20 mM FeCl_3_ in a volumetric ratio of 10:1:1. For the assay, 0.1 mL of the extract was mixed with 4.9 mL of the FRAP reagent and incubated at 37 °C for 30 min. The resulting absorbance was recorded at 593 nm using a spectrophotometer. Antioxidant activity was calculated based on a trolox standard curve and reported as micromoles of trolox equivalents per gram of dry weight (μmol_TE/g_DW).

### 2.6. Determination of α-Glucosidase Inhibitory Activity

The inhibitory activity of *A. trifoliata* fruit extracts against α-glucosidase was evaluated according to the protocol described by [25]. The reaction system contained 50 μL of the sample extract and 50 μL of α-glucosidase enzyme solution (0.2 U/mL) (S10050, Shanghai yuanye Bio-Technology Co., Ltd., Shanghai, China). The mixture was incubated at 37 °C for 10 min. Subsequently, 100 μL of 3 mM p-nitrophenyl-α-D-glucopyranoside (PNPG) was added as the enzymatic substrate, and the reaction was continued for another 10 min at the same temperature. The enzymatic activity was stopped by adding 750 μL of 0.1 M sodium carbonate (Na_2_CO_3_). Absorbance was measured at 405 nm using a spectrophotometer. Acarbose served as the positive control. The percentage of α-glucosidase inhibition was calculated using the following equation:

$$I\;(\%)\;=\;1-\frac{A1-A2}{A3-A4}\times 100\%$$

where *A*1 represents the absorbance of the sample containing both enzyme and substrate, *A*2 is the absorbance of the sample without the enzyme, *A*3 denotes the absorbance of the control containing enzyme and substrate, and *A*4 corresponds to the absorbance of the control without the enzyme. The IC_50_ value (concentration required to inhibit 50% of enzyme activity) was calculated based on the inhibition percentages of five different extract concentrations (0.2, 0.5, 1.0, 2.0, 5.0 mg/mL).

### 2.7. UPLC-MS/MS Metabolomics Analysis

#### 2.7.1. UPLC Conditions

Metabolite separation was carried out using a UPLC-ESI-MS/MS system comprising an ExionLC™ AD ultra-performance liquid chromatography module coupled with a SCIEX TripleTOF 6600 (SCIEX, Singapore; Resolution: ≥35,000 (FWHM); Mass accuracy: <1 ppm) triple quadrupole mass spectrometer. Chromatographic separation was achieved using an Agilent SB-C18 column (2.1 × 100 mm, 1.8 μm particle size). The mobile phase included solvent A (water containing 0.1% (*v*/*v*) formic acid) and solvent B (acetonitrile with 0.1% (*v*/*v*) formic acid). A gradient elution was applied as follows: 95% A at 0 min, a linear gradient to 5% A by 9.0 min, maintained until 10.0 min, then returned to initial conditions by 11.1 min, and held until 14.0 min. The flow rate was maintained at 0.35 mL/min, the column temperature was set at 40 °C, and 2 µL of sample extract was injected for each run. Simultaneously, quality control (QC) samples were prepared by mixing aliquots of all extracts.

#### 2.7.2. ESI-Q TRAP-MS/MS

Mass spectrometry detection was performed using a TripleTOF 6600+ system (SCIEX, Singapore) fitted with an electrospray ionization (ESI) source in both positive and negative ionization mode. The system was operated through Analyst 1.6.3 software. The ion source parameters were configured as follows: an ion spray voltage of 5500 V (positive ion mode), −4000 V (negative ion mode), 550 °C ion source temperature, curtain gas at 25 psi, ion source gas 1 at 50 psi, and gas 2 at 60 psi. The collision-activated dissociation (CAD) was set to high, the QQQ scan was obtained by the MRM mode, and the collision gas (nitrogen) set at medium.

Data acquisition was conducted in multiple reaction monitoring (MRM) mode, with each metabolite monitored via optimized precursor-to-product ion transitions. Instrument settings such as declustering potential (DP) and collision energy (CE) were fine-tuned for each metabolite to ensure optimal sensitivity. The MRM scheduling was aligned with chromatographic retention times to enhance detection efficiency. The MetWare database (MWDB) and multi-reaction monitoring (MRM) served as the foundation for the qualitative and quantitative mass spectrometry analysis of metabolites in samples. The process of metabolite identification involves intelligently matching each metabolite’s secondary spectrum and RT in the sample to those in the MWDB database. The MS and MS2 tolerances were set at 20 ppm; RT tolerance was set at 0.2 min. Level indicates the level of substance identification: Level 1 > Level 2 > Level 3, with accuracy decreasing in sequence. Level 1 has the highest accuracy. Level 1: the secondary mass spectrometry of the sample substance (all fragment ions of the substance)—RT and the matching score of the database substance > 0.7. Level 2: the secondary mass spectrometry of the sample substance (all fragment ions of the substance)—RT and the matching score of the database substance 0.5–0.7; Level 3: The Q1, Q3, RT, DP, and CE of the sample substances were verified to be consistent with those in the database.

### 2.8. Metabolite Identification and Quantification

Metabolites were identified by comparing precursor and product ion pairs (Q1 and Q3), retention times (RT), and fragmentation patterns against the MetWare database. Redundant signals—including isotopes, adduct ions (e.g., K^+^, Na^+^, NH_4_^+^), and fragment ions of larger molecules—were filtered to improve annotation accuracy.

Quantification was based on peak area integration in MRM mode using QQQ detection. Peak areas were normalized and aligned across samples, and only consistent signals were used for further statistical analysis [26]. To initially remove interference, the quadrupole rod in the MRM mode first filters the target substance’s parent ions, and rejects ions that correspond to other compounds with differing molecular weights. Following ionization, the precursor ions exit the collision chamber to create a large number of fragment ions. These are then filtered by QQQ to choose a desired characteristic fragment ion, removing non-target ion interference for a more precise and repeatable measurement. After the metabolite mass spectrometry data of various samples were obtained, the mass spectrum peaks of the same metabolites in the various samples were integrated and corrected after the peak area was integrated to reveal the mass spectrum peaks of all the substances.

### 2.9. Statistical Analysis

All experimental data were generated from three independent biological replicates, unless otherwise specified. Statistical differences among groups were assessed using one-way analysis of variance (ANOVA), followed by the least significant difference (LSD) post hoc test, with significance determined at *p* < 0.05. Analyses were conducted using SPSS software version 23.0 (IBM, Armonk, NY, USA), and the outcomes are expressed as means ± standard error (SE).

For metabolomic datasets, raw mass spectrometry signals were processed using Analyst software version 1.6.3 (AB Sciex). Multivariate statistical tools—such as principal component analysis (PCA), hierarchical clustering (HCA), orthogonal partial least squares discriminant analysis (OPLS-DA), and K-means clustering—were applied through R statistical software (Version 4.1.2) (https://www.r-project.org/). Differential metabolites were screened based on variable importance in projection (VIP) values ≥ 1 and absolute log_2_ fold change ≥ 1.

To interpret biological significance, functional annotation and enrichment of the identified differential metabolites were performed using the KEGG database (https://www.kegg.jp/kegg/pathway.html, accessed on 10 July 2025). Differential metabolite enrichment analysis of the KEGG pathway was performed using metabolite set enrichment analysis (MSEA). The hypergeometric test’s *p*-values were used for determining the statistical significance of enrichment analysis.

The most enriched pathways were further visualized using bubble plots, and cumulative metabolite changes within pathways were evaluated using differential abundance scoring.

## 3. Results

### 3.1. Morphological Characterization of Fruits

**Figure 1:**
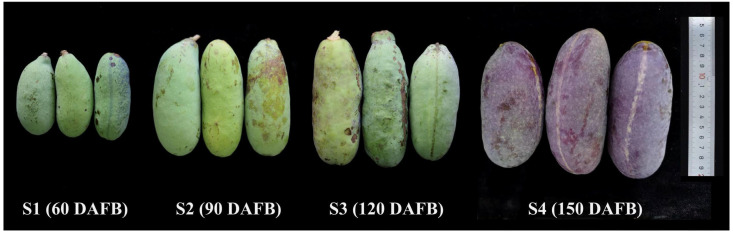
The fruit of *A. trifoliata* at different growth stages. (The unit of the ruler is centimeters).

Morphological characteristics of *A. trifoliata* fruit at different growth stages are shown in Figure 1 and Table 1. All of the SFW, FL, and FD increased significantly with fruit growth. The SFW increased significantly from 75.50 ± 1.15 g at S1 stage to 287.07 ± 34.37 g at S4 stage. The growth trends in the FL and FD from S1 stage to S4 stage were basically the same, increasing from 83.28 ± 1.03 mm to 148.25 ± 2.85 mm and 47.63 ± 1.23 mm to 56.87 ± 1.25 mm, respectively. For the DM, the values of DM increased from 19.46 ± 0.73% at S1 stage to 30.18 ± 1.31% at S3 stage, but decreased at S4 stage (26.19 ± 1.23%).

### 3.2. Dynamic Changes in Total Phenolics, Total Flavonoids, Antioxidant Activities, and α-Glucosidase Inhibitory Activity at Different Fruit Growth Stages

The variation in antioxidant components of *A. trifoliata* fruits across different developmental stages is summarized in Table 2. Both total phenolic content (TPC) and total flavonoid content (TFC) exhibited a consistent declining trend as the fruit matured. The highest levels were recorded at the earliest stage (S1), with 28.20 ± 0.81 mg GAE/g dry weight for TPC and 63.43 ± 2.51 mg RE/g dry weight for TFC. These values progressively decreased throughout development, reaching their lowest at the fully ripe stage (S4), with TPC and TFC values of 1.85 ± 0.24 mg GAE/g and 4.61 ± 0.13 mg RE/g, respectively.

ABTS, DPPH, and FRAP assays were performed to evaluate the antioxidant activities of *A. trifoliata* fruit at different growth stages (Table 2). The radical cation ABTS scavenging activities and DPPH radical scavenging activities showed the same trend during fruit development. That is, both of them have the maximum values at the S1 stage (97.88% and 90.09%, respectively), and then dropped sharply with the fruit ripening (15.34% and 5.56% at the S4 stage, respectively). The FRAP showed a similar trend with ABTS and DPPH, but there was no significant difference between the S3 and S4 stages. The α-glucosidase inhibitory activity decreased continuously with fruit maturation, with IC_50_ values of 0.81 ± 0.11, 2.82 ± 0.57, 10.89 ± 0.72, and 11.45 ± 2.16 mg/mL, respectively.

### 3.3. Metabolite Profiles of A. trifoliata Fruit

The qualitative and quantitative analyses of metabolic components in *A. trifoliata* fruit at different growth stages were performed by using UPLC-MS/MS. Total ion current (TIC) diagrams and multi-peak detection plots of one quality control (QC) sample are shown in Supplementary Figure S1. Overlay analysis of the TIC QC and sample multi-peak detection diagrams (Supplementary Figure S2) revealed good repeatability and reliability of the data detected by MS. A total of 1595 metabolites were detected (Supplementary Table S1), including 329 terpenoids (20.63%), 223 amino acids and derivatives (13.98%), 188 phenolic acids (11.79%), 187 flavonoids (11.72%), 140 lipids (8.78%), 107 alkaloids (6.71%), 82 organic acids (5.14%), 65 lignans and coumarins (4.08%), 59 nucleotides and derivatives (3.7%), 11 tannins (0.69%), 10 quinones (0.63%), 6 steroids (0.38%), and 188 others (11.79%) (Supplementary Figure S3).

### 3.4. Multivariate Statistical Analysis of Metabolites

**Figure 2:**
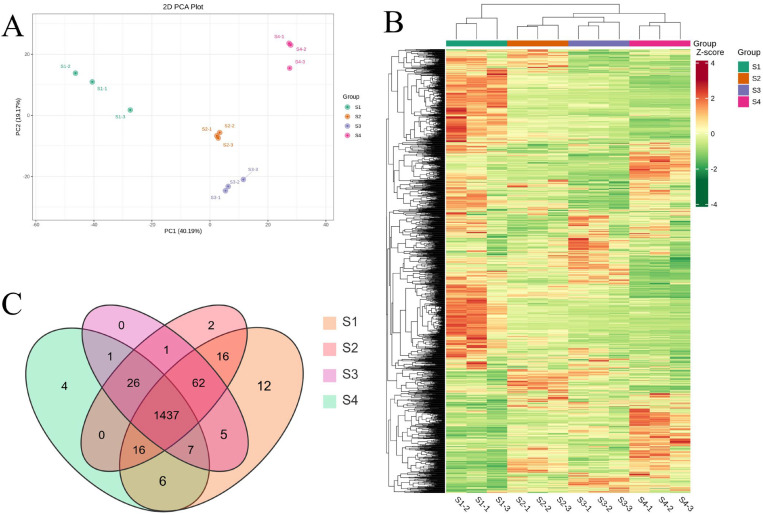
An analytical overview of the metabolites detected in the four growth stages of *A. trifoliata* fruit. (**A**) PCA score plot. (**B**) Clustered heatmap of all metabolites. (**C**) Venn diagram of metabolite distribution in different stages.

Principal component analysis (PCA) was performed on all 1595 detected metabolites to assess variation across developmental stages. As illustrated in Figure 2A, PC1 and PC2 accounted for 40.19% and 19.17% of the total variance, respectively. The three biological replicates at each stage clustered closely together, indicating high within-group consistency and clear metabolic distinctions among stages. Hierarchical clustering analysis (HCA) further supported these findings, with the heatmap (Figure 2B) showing distinct metabolite compositions across stages and strong similarity among replicates. Notably, S2 and S3 samples clustered more closely, suggesting similar metabolic profiles during mid-development. Overall, both PCA and HCA results delineated the fruit developmental process into three main metabolic phases: early (S1), middle (S2–S3), and late (S4). Additionally, Venn diagram analysis revealed that 1437 metabolites were shared across all stages, with few stage-specific compounds, highlighting a core set of conserved metabolites throughout development (Figure 2C).

**Figure 3:**
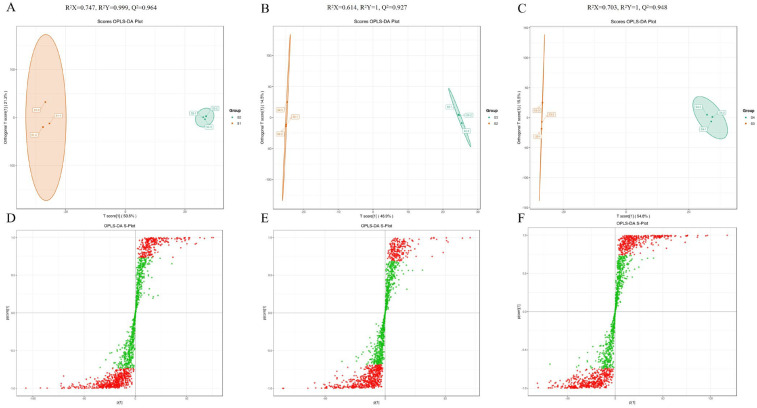
Orthogonal partial least squares discriminant analysis (OPLS-DA) scores. Scores of the OPLS-DA model with (**A**) S2 vs. S1, (**B**) S3 vs. S2, and (**C**) S4 vs. S3. OPLS-DA s-plot model with (**D**) S2 vs. S1, (**E**) S3 vs. S2, and (**F**) S4 vs. S3. (Red dots indicated that the VIP values of these metabolites were greater than 1, while green dots indicated that the VIP values of these metabolites were less than or equal to 1).

In this study, we used OPLS-DA models to further compare the metabolite differences between stages (Figure 3; Supplementary Figure S4). The Q2 values of all models exceeded 0.9, demonstrating that these models were reliable. In the OPLS-DA models, samples extracted from different stages were all clearly separated, indicating that the metabolite phenotype changed significantly during the four stages. Contrary to the results of PCA and HCA, the S2 and S3 samples were clearly separated in the OPLS-DA model.

### 3.5. Differential Metabolite Screening

**Figure 4:**
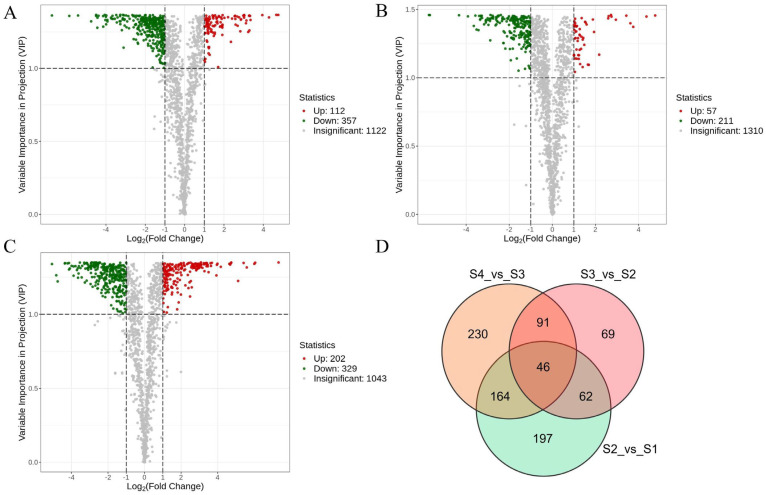
Differential metabolite analysis of *A. trifoliata* fruit at different growth stages. (**A**–**C**) Volcano plots of differential metabolites in different pairwise comparisons: (**A**) S2 vs. S1, (**B**) S3 vs. S2, and (**C**) S4 vs. S3. Green dots indicate downregulated, differentially expressed metabolites; red dots indicate upregulated, differentially expressed metabolites; and gray dots indicate detected metabolites with insignificant differences in expression. (**D**) Venn diagram showing the common and unique metabolites in the comparison groups.

Differential metabolites between consecutive developmental stages were identified using thresholds of fold change (FC ≥ 2 or ≤0.5) and variable importance in projection (VIP ≥ 1). The number of upregulated and downregulated metabolites from each pairwise comparison is shown in Figure 4 (Supplementary Figure S5; Supplementary Table S2). A total of 469 differential metabolites were detected between S2 and S1, including 112 upregulated and 357 downregulated compounds. Between S3 and S2, 268 differential metabolites were identified (57 upregulated, 211 downregulated), while the S4 vs. S3 comparison revealed 531 differentially accumulated metabolites (202 upregulated, 329 downregulated). Venn diagram analysis revealed that only 46 metabolites were shared across all three comparisons, whereas 197, 69, and 230 metabolites were uniquely altered in the S2 vs. S1, S3 vs. S2, and S4 vs. S3 comparisons, respectively. These results suggest that the majority of differential metabolites are stage-based transformation, reflecting distinct metabolic shifts at each developmental transition.

### 3.6. Dynamics of the Different Metabolites During Fruit Maturation

**Figure 5:**
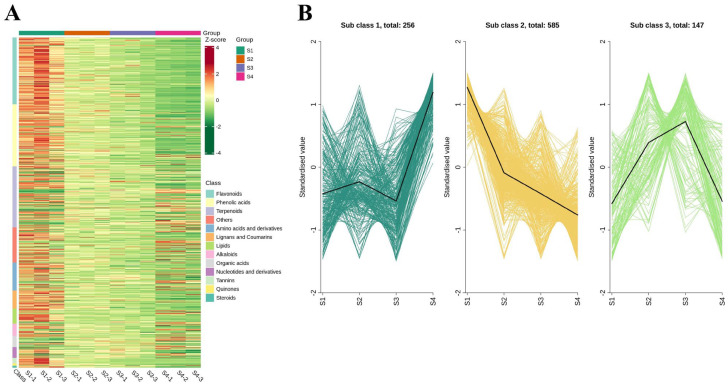
HCA (**A**) and K-means (**B**) cluster analysis revealing the dynamic changes in differential metabolites across the four studied growth stages in *A. trifoliata* fruit.

To investigate the dynamic variation patterns of metabolites during *A. trifoliata* fruit maturation, both hierarchical clustering and K-means clustering analyses were conducted on the 988 differentially accumulated metabolites (Figure 5). The heatmap (Figure 5A) revealed a general decline in the abundance of flavonoids and phenolic acids as fruit development progressed. K-means clustering further categorized these metabolites into three distinct subclusters based on their accumulation trends across the four developmental stages (Figure 5B), providing insight into stage-specific metabolic reprogramming during fruit ripening. Subclass 1 contained 256 metabolites whose contents fluctuated in S1-S3 stages (Table S3a), and dramatically increased at the ripening stage of fruit. The representative metabolites in this class included amino acids and their derivatives, terpenoids, and phenolic acids. Although Subclass 2 has the greatest abundance of metabolites, 585 differential metabolites (Table S3b), the compounds in this group constantly declined throughout the whole growth period and reached their lowest levels at the ripening stage. The representative metabolites in Subclass 2 included flavonoids, phenolic acids, and terpenoids. For Subclass 3, 147 metabolites reached their accumulation plateau at S3 stage, while dropping sharply at ripening stage (Table S3c). The representative metabolites in this group included terpenoids and some amino acids and their derivatives.

### 3.7. Functional Oligopeptide Prediction in A. trifoliata Fruit

The functional oligopeptides in differential metabolites were determined for the four studied growth stages in *A. trifoliata* fruit (Table S3). This analysis was based on searching for the oligopeptide sequences in the BIOPEP database to obtain the oligopeptide activity information [27]. The functional activity of 23 oligopeptides is noted in Table 3. Subclass 1 contained 7 functional oligopeptides with multiple activities, including ACE-inhibiting, anti-inflammatory, antioxidative, and antidiabetic properties. Subclass 2 had 13 oligopeptides, the largest number of functional oligopeptides in the three subclasses, with AEL, AY, GF, GY, LP, MYY, and VW having ACE-inhibiting activity. Three functional oligopeptides (DE, LF, KG) were detected in the Subclass 3, which provide ACE-inhibiting, antidiabetic, and glutamate carboxypeptidase II-inhibiting activities.

### 3.8. KEGG Annotation and Enrichment Analysis of Differential Metabolism

**Figure 6:**
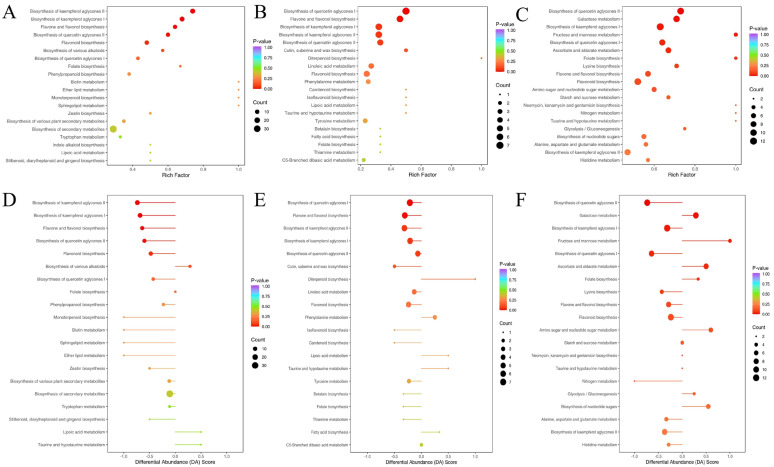
KEGG enrichment maps of differential metabolites in different pairwise comparisons: (**A**) S2 vs. S1, (**B**) S3 vs. S2, and (**C**) S4 vs. S3. (**A**–**C**) The abscissa represents the enrichment factor of the pathway and the ordinate shows the names of pathways. The dot color represents the *p*-value, with darker red indicating stronger enrichment effects. The dot size represents the number of metabolites enriched in the pathways. (**D**–**F**) The DA score reflects the overall change in all metabolites in a metabolic pathway (DA = (number of upregulated differential metabolites − number of downregulated differential metabolites)/number of all metabolites annotated in this pathway).

To investigate the functional implications of metabolic changes during *A. trifoliata* fruit development, KEGG pathway enrichment analysis was performed on the differentially accumulated metabolites identified between consecutive stages. A total of 96 differential metabolites between S1 and S2 were mapped to 69 pathways, 53 metabolites between S2 and S3 were assigned to 47 pathways, and 126 metabolites between S3 and S4 were distributed across 82 pathways (Table S4). Among these, the most frequently enriched categories were general metabolic pathways (ko01100) and secondary metabolite biosynthesis (ko01110), with relative frequencies of 41.67%, 45.28%, and 59.52% for metabolic pathways, and 36.46%, 35.85%, and 36.51% for secondary metabolism in S2 vs. S1, S3 vs. S2, and S4 vs. S3, respectively. Major enriched pathways are illustrated using bubble plots (Figure 6A–C).

Specifically, in the S2 vs. S1 comparison, significant enrichment was observed in the biosynthesis of kaempferol aglycones I and II (MetMap113 and MetMap114), quercetin aglycones II (MetMap116), flavone and flavonol biosynthesis (ko00944), and general flavonoid biosynthesis (ko00941). In the transition from S2 to S3, enrichment was mainly found in quercetin aglycone I biosynthesis (MetMap115) and flavone/flavonol biosynthesis (ko00944). For the S4 vs. S3 stage, enriched pathways included galactose metabolism (ko00052), fructose and mannose metabolism (ko00051), ascorbate and aldarate metabolism (ko00053), folate biosynthesis (ko00790), and various branches of kaempferol and quercetin biosynthesis. Notably, the flavone and flavonol biosynthesis pathway (ko00944) showed highly significant enrichment (*p* < 0.01) during the early developmental transitions (S2 vs. S1 and S3 vs. S2).

To visualize broader pathway-level trends, differential abundance scores (DA scores) were calculated to assess cumulative changes within each metabolic pathway. The top 20 significantly enriched pathways, based on *p*-values, are shown in Figure 6D–F. Flavonoid-related pathways—including kaempferol, quercetin, and flavonol biosynthesis—were predominantly enriched and downregulated during early developmental stages (S1–S3). In contrast, sugar metabolism pathways such as galactose metabolism and fructose/mannose metabolism were enriched and upregulated during the ripening phase (S3–S4), reflecting metabolic shifts associated with fruit softening and sweetness accumulation.

### 3.9. Correlation Analysis of Secondary Metabolites in A. trifoliata and Antioxidant Activity, α-Glucosidase Inhibitory Activity

**Figure 7:**
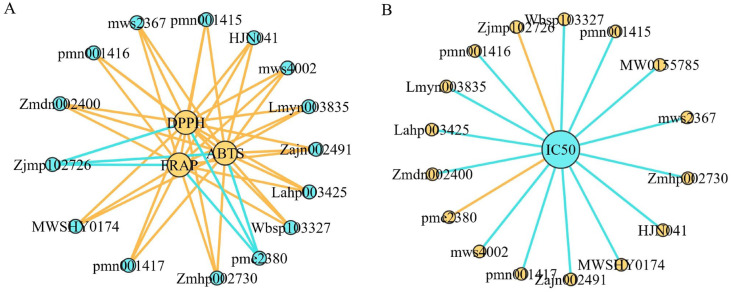
Network diagram between antioxidant capacity and metabolites (**A**), IC_50_ and metabolites (**B**). The number above the circle indicates the ingredient ID of Metware, the line connecting the two circles represents the correlation (r ≥ 0.8, *p* < 0.01), and yellow lines represent a positive correlation and the blue lines represent a negative correlation.

To gain deeper insight into the key antioxidant components in *A. trifoliata*, we conducted Spearman correlation analysis between differential metabolites identified across consecutive developmental stages (Table S2) and the measured antioxidant activities (DPPH, ABTS, and FRAP). A total of 13 metabolites exhibited strong positive correlations with all three antioxidant assays (r ≥ 0.8, *p* < 0.01; Figure 7A). These included nine flavonoids, two phenolic acids, one alkaloid, and one terpenoid, indicating their substantial contribution to the antioxidant potential of *A. trifoliata* fruit.

Additionally, correlation analysis was performed between differential metabolites and the IC_50_ values of α-glucosidase inhibitory activity. Since lower IC_50_ values reflect stronger inhibition, metabolites that negatively correlated with IC_50_ are considered critical contributors to this bioactivity. In total, 14 metabolites showed a significant negative correlation with IC_50_ values (r ≥ 0.8, *p* < 0.01; Figure 7B), comprising nine flavonoids, two phenolic acids, one alkaloid, one terpenoid, and one amino acid derivative. These findings highlight the dual role of several metabolites—particularly flavonoids and phenolic acids—in contributing to both antioxidant and α-glucosidase inhibitory activities during fruit development.

## 4. Discussion

The developmental stage of *A. trifoliata* plays a critical role in shaping its metabolic composition and biological activity. While prior studies have characterized specific compounds such as triterpenoids and saponins using conventional phytochemical approaches [6,28,29,30], few metabolomics approaches have been used to investigate the similarities and differences in the metabolites present in *A. trifoliata* fruit at different stages of development. In this study, we used a widely targeted metabolomics technique to analyze the metabolites present in *A. trifoliata* fruit at different stages of development. This revealed the accumulation patterns and metabolic networks involved in the biosynthesis of secondary metabolites and functional components, thereby providing a scientific basis for exploiting *A. trifoliata* resources.

This study demonstrated that the developmental stage significantly affects the morphological characteristics of *A. trifoliata* fruit. An increase in fruit size (fruit weight, fruit length, and fruit diameter) was associated with fruit ripening, consistent with previous reports for *A. trifoliata* fruit [3] (Zou et al., 2022). However, the dry matter value of *A. trifoliata* fruit increased continuously from the S1 to S3 stage, and then declined at the S4 stage, mainly due to the degradation of substances such as starch caused by ripening [31] (Nazir et al., 2024).

Phenolic compounds and flavonoids are important secondary metabolites that can protect cells from oxidative damage, increase plant resistance to pathogens, and provide antioxidants for the human diet [32,33,34]. In this study, the results showed that the total phenolic and total flavonoid levels declined with the advancement of *A. trifoliata* fruit development. Such variations in the content of total phenolics and flavonoids were also reported in our previous study [3] (Zou et al., 2022) and in many other fruit crops, including pomegranate, red raspberry, Brazilian cherry, blackberry, strawberry, and acerola [35,36,37,38,39,40]. Although some influencing factors such as light intensity, structural genes, and temperature have been reported to influence the biosynthesis of phenolic compounds and flavonoids [41,42], the results of this study clearly demonstrate that the developmental stage is one of the primary variables affecting the accumulation of secondary metabolites in *A. trifoliata* fruit. The decrease in phenolics and flavonoids content in *A. trifoliata* fruit may be due to a reduction in phenolics synthesis or the continuous transformation of phenols into other substances during development. Concurrently, as the weight of the fruit increases, the phenolics and flavonoids content per unit mass decreases [43,44]. Furthermore, three different methods (ABTS, DPPH, and FRAP) were used to evaluate the antioxidant activity of *A. trifoliata* fruit at different developmental stages, and the results are presented in Table 2. Clearly shown is that the antioxidant activity of *A. trifoliata* fruit declined with fruit development, which is consistent with the dynamic changing trend in total phenolics and flavonoids. Additionally, the α-glucosidase inhibitory activity of *A. trifoliata* fruit also showed a similar trend with total phenolics and flavonoids. These results proved that the developmental stage is one of the primary variables affecting the antioxidant and α-glucosidase inhibitory activities in *A. trifoliata* fruit.

Interestingly, the high accumulation of flavonoids and phenolic acids in early stages aligns with the strong enrichment of flavonoid biosynthesis pathways (ko00941, ko00944), which subsequently decline as sugar-related pathways (e.g., galactose and fructose metabolism) become more active during ripening. This metabolic shift reflects a strategic redirection of carbon flow—from defense-oriented secondary metabolites to energy-rich primary metabolites for fruit maturation and palatability [45].

The structural diversity of flavonoids, particularly those derived from quercetin and kaempferol, highlights their glycosylation patterns as modulators of solubility and biological activity [46,47]. Kaempferol-derived substances included Kaempferol-3-O-rutinoside-7-O-glucoside, Kaempferol-4′-O-glucoside*, Kaempferol-7-O-glucoside, Kaempferol-3-O-rhamnoside, Kaempferol-3-O-glucoside-7-O-rhamnoside*, and Kaempferol-3-O-galactoside-4′-O-glucoside. Quercetin-derived substances included those such as Quercetin-3-O-glucoside-7-O-xyloside, Quercetin-3-O-arabinoside, Quercetin-3,7-Di-O-glucoside*, Quercetin-3-O-glucoside (Isoquercitrin)*, Quercetin-3-O-rhamnoside (Quercitrin), and Quercetin-7-O-glucoside. Glycosylation contributes to the structural diversity and complexity of flavonoids, improving water solubility and stability, and altering bioactivity and bioavailability in vivo [47,48]. For example, Quercetin-3-O-arabinoside has a significant effect on α-glucosidase inhibition that could be exploited to develop hypoglycemic nutraceuticals [49]. Therefore, the abundant flavonoids identified in *A. trifoliata* fruit will be an essential reference for the isolation and study of unique flavonoids in *A. trifoliata*.

A particularly novel insight from this study is the identification of functional oligopeptides enriched during late fruit development. Functional groups of the identified oligopeptides in *A. trifoliata* fruit include ACE-inhibiting, anti-inflammatory, antioxidative, antidiabetic, hypouricemic and hypolipidemic oligopeptides. For example, the use of ACE-inhibiting oligopeptides have been reported in the management of several diseases, including cardiovascular disease, increased blood pressure, type 2 diabetes, and obesity [50,51,52]. Indeed, various phytochemical components have been isolated and reported from various parts of *A. trifoliata*, including triterpenoids, triterpenoid saponins, polyphenols, and alkaloids, which provide diuretic, analgesic, anti-inflammatory, antibacterial, and antioxidative properties [20,53,54,55,56,57]. This study first detected and annotated many functional oligopeptides with multiple activities. Annotated peptides such as AEL, AY, and GF possess antihypertensive, antioxidative, and antidiabetic properties [51], implying new potential bioactivities beyond those historically associated with *A. trifoliata* [4,20]. These findings broaden the pharmacological landscape of this fruit, warranting future research into peptide biosynthesis and stability.

Moreover, the integration of metabolite–bioactivity correlation analysis highlights key compounds—particularly flavonoids and phenolic acids—as central players in both antioxidant and α-glucosidase inhibitory mechanisms. Compounds such as salidroside and catechins have been widely documented for their multifunctional therapeutic properties, including anti-inflammatory, anti-aging, and antidiabetic effects [58,59,60,61,62]. Their consistent correlation with measured activities supports their biomarker potential for quality evaluation and functional product development.

In summary, *A. trifoliata* exhibits a dynamic and stage-specific metabolic landscape, with early development favoring antioxidant-rich phenolics and flavonoids, and maturation stages characterized by bioactive peptides and sugar metabolism. This temporal metabolic programming not only underpins fruit quality traits, but also informs optimal harvest timing for different end uses—whether medicinal, nutritional, or industrial. According to the metabolic profiles of *A. trifoliata* fruit, we recommend harvesting it at an early development stage for medicinal use, and consuming it as fruit when it is fully mature. These findings lay a foundation for precision breeding, targeted utilization, and deeper exploration of the molecular pathways governing metabolite biosynthesis in *A. trifoliata*.

## 5. Conclusions

This study provides a comprehensive overview of the dynamic changes in secondary metabolites and functional compounds during the development of *A. trifoliata* fruit using widely targeted metabolomics. A total of 1595 metabolites were identified, with significant shifts in flavonoids, phenolic acids, terpenoids, and oligopeptides across developmental stages. Early-stage fruits are rich in antioxidant and α-glucosidase inhibitory compounds, while ripe fruits accumulate bioactive oligopeptides with potential health-promoting properties. Pathway enrichment revealed a developmental transition from flavonoid biosynthesis to sugar metabolism, reflecting functional shifts in fruit physiology. Correlation analyses further identified key metabolites closely linked to antioxidant and enzyme inhibitory activities, offering potential biomarkers for quality evaluation. Overall, these findings highlight stage-specific metabolic signatures that can inform the optimized harvesting and targeted utilization of *A. trifoliata* for both medicinal and nutritional applications. Future work should focus on functional validation of key metabolites and elucidation of their biosynthetic regulation.

## Figures and Tables

**Table 1:** Morphological characteristics of *A. trifoliata* fruit at different growth stages.

Stages	Single Fruit Weight/g	Fruit Length/mm	Fruit Diameter/mm	Dry Matter/%
S1	75.50 ± 1.15 d	83.28 ± 1.03 c	47.63 ± 1.23 c	19.46 ± 0.73 c
S2	161.11 ± 16.41 c	115.81 ± 5.86 b	47.77 ± 1.09 c	23.02 ± 1.80 bc
S3	203.31 ± 11.28 b	123.87 ± 7.04 b	52.74 ± 0.71 b	30.18 ± 1.31 a
S4	287.07 ± 34.37 a	148.25 ± 2.85 a	56.87 ± 1.25 a	26.19 ± 1.23 ab

Means with different letters within the same column indicate statistical differences at the *p* < 0.05 level. S1: 60 DAFB (days after full bloom), S2: 90 DAFB, S3: 120 DAFB, S4: 150 DAFB.

**Table 2:** Total phenolics, total flavonoids, antioxidant activities, and IC50 of α-glucosidase inhibitory activity of *A. trifoliata* fruit at different growth stages.

Stages	Total Phenolics (GAE_mg/g_DW)	Total Flavonoids (RE_mg/g_DW)	ABTS (%)	DPPH (%)	FRAP (μmol_Trolox/g_DW)	IC50 (mg/mL_DW)
S1	28.20 ± 0.81 a	63.43 ± 2.51 a	97.88 ± 0.86 a	90.09 ± 0.35 a	164.66 ± 4.24 a	0.81 ± 0.11 c
S2	6.85 ± 0.78 b	13.84 ± 0.71 b	33.35 ± 2.68 b	20.68 ± 0.65 b	44.90 ± 3.86 b	2.82 ± 0.57 b
S3	2.99 ± 0.56 c	7.86 ± 0.54 c	20.47 ± 1.31 c	8.70 ± 0.65 c	11.58 ± 1.89 c	10.89 ± 0.72 a
S4	1.85 ± 0.24 c	4.61 ± 0.13 c	15.34 ± 0.52 d	5.56 ± 0.27 d	11.99 ± 0.04 c	11.45 ± 2.16 a

Means with different letters within the same column indicate statistical differences at the *p* < 0.05 level.

**Table 3:** Functional oligopeptides predicted in *A. trifoliata* fruit.

Subclass	Oligopeptide Sequences	Activity
Subclass 1	ER	ACE inhibitor; neprilysin inhibitor; hypotensive
	GW	ACE inhibitor; antioxidative; inhibitor of tripeptidyl peptidase II; dipeptidyl peptidase IV inhibitor
	KF	ACE inhibitor; renin inhibitor; CaMPDE inhibitor; dipeptidyl peptidase IV inhibitor
	KY	ACE inhibitor; dipeptidyl peptidase IV inhibitor
	MH	dipeptidyl peptidase IV inhibitor
	FH	hypouricemic
	FT	renin inhibitor
		
Subclass 2	AEL	ACE inhibitor
	AY	ACE inhibitor; tubulin-tyrosine ligase inhibitor; antioxidative; dipeptidyl peptidase IV inhibitor
	DWR	alpha-amylase inhibitor; alpha-glucosidase inhibitor
	EP	dipeptidyl peptidase IV inhibitor
	EYW	antioxidative
	GF	ACE inhibitor; dipeptidyl peptidase III inhibitor; acylaminoacyl peptidase inhibitor; dipeptidyl peptidase IV inhibitor; inhibitor of tripeptidyl peptidase II
	GY	ACE inhibitor; dipeptidyl peptidase IV inhibitor; inhibitor of tripeptidyl peptidase II
	LP	ACE inhibitor; lactocepin inhibitor; dipeptidyl peptidase IV inhibitor
	MYY	ACE inhibitor; antioxidative
	PYY	antioxidative
	YPY	alpha-glucosidase inhibitor; dipeptidyl peptidase IV inhibitor
	VL	stimulating; dipeptidyl peptidase IV inhibitor
	VW	ACE inhibitor; antioxidative; alpha-glucosidase inhibitor; dipeptidyl peptidase IV inhibitor; hypouricemic
		
Subclass 3	DE	glutamate carboxypeptidase II inhibitor
	LF	ACE inhibitor
	KG	ACE inhibitor; dipeptidyl peptidase IV inhibitor

## Data Availability

The original contributions of this study are detailed within the manuscript. Further inquiries should be directed to the corresponding author.

## Supplementary Materials

The following supporting information can be downloaded at: https://www.mdpi.com/article/10.3390/antiox14091039/s1, Figure S1. Total ion current diagram of the quality control samples in (A) electrospray ionization (ESI)+ and (B) in ESI– mode. Multi-peak detection plot of the metabolites in multiple reaction monitoring (MRM) in (C) ESI+ mode, and (D) ESI– mode; Figure S2. Total ion current overlaps of the three quality control samples using mass spectrometry detection. (A) Total ion current (TIC) overlay plot in electrospray ionization (ESI)+ mode. (B) TIC overlay plot in (ESI)– mode; Figure S3. Metabolite classes and quantities detected in the samples from the four growth stages of *A. trifoliata* fruit; Figure S4. Orthogonal partial least squares discriminant analysis (OPLS-DA) scores. Scores of the OPLS-DA model with (A) S3 vs. S1, (B) S4 vs. S1, and (C) S4 vs. S2. OPLS-DA s-plot model with (D) S3 vs. S1, (E) S4 vs. S1, and (F) S4 vs. S2. Figure S5. Differential metabolite analysis of *A. trifoliata* fruit at different growth stages. (A–C) Volcano plots of differential metabolites in different pairwise comparisons: (A) S3 vs. S1, (B) S4 vs. S1, and (C) S4 vs. S2. Green dots indicate downregulated, differentially expressed metabolites; red dots indicate upregulated, differentially expressed metabolites; and gray dots indicate detected metabolites with insignificant differences in expression. (D) Venn diagram showing the common and unique metabolites in the comparison groups; Table S1: A total of 1595 metabolites were characterized in four growth stages (S1–S4) of *A. trifoliata* fruit; Table S2a: Significant compounds in the comparison S2 vs. S1; Table S2b: Significant compounds in the comparison S3 vs. S2; Table S2c: Significant compounds in the comparison S4 vs. S3; Table S2d: Significant compounds in the comparison S3 vs. S1; Table S2e: Significant compounds in the comparison S4 vs. S1; Table S2f: Significant compounds in the comparison S4 vs. S2; Table S3a: The 256 metabolites in Subclass1; Table S3b: The 585 metabolites in Subclass2; Table S3c: The 147 metabolites in Subclass3; Table S4: KEGG classification of the differential metabolites in the comparison S4 vs. S3.
