# Supplementary material for: Widely Targeted Metabolomics Analysis Reveals Developmental Shifts in Antioxidants and Functional Peptides in Akebia trifoliata
Source: Antioxidants (Basel). 2025 Aug 24;14(9):1039. doi: 10.3390/antiox14091039 (PMC12466582; doi:10.3390/antiox14091039)
Supplement: Supplementary file 1 [file antioxidants-14-01039-s001.zip › Figures S1-S5.pdf]

Supplementary Material

1 Supplementary Figures

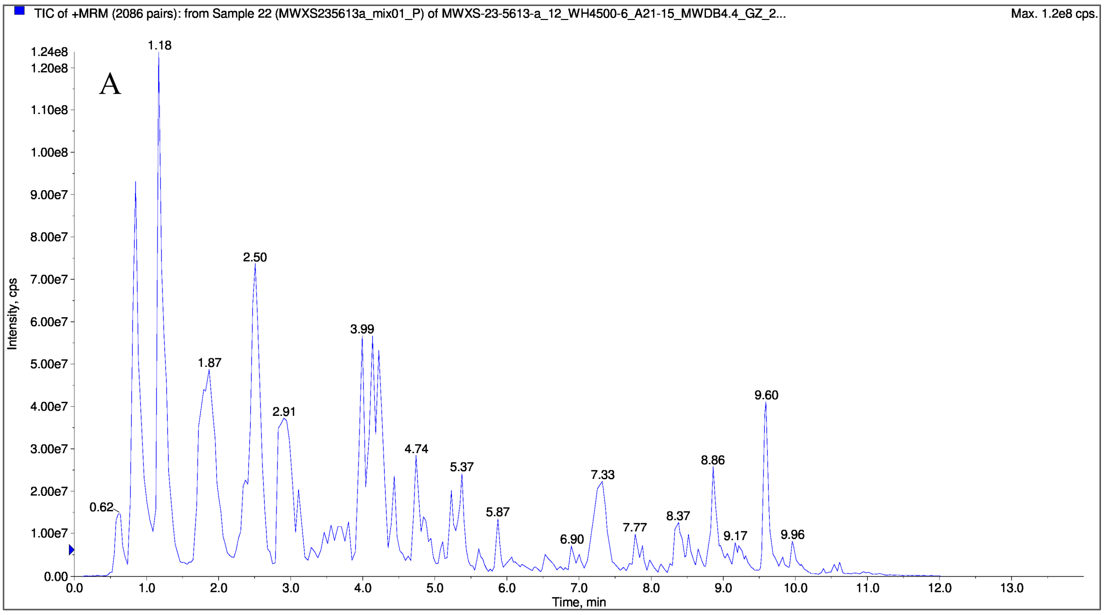

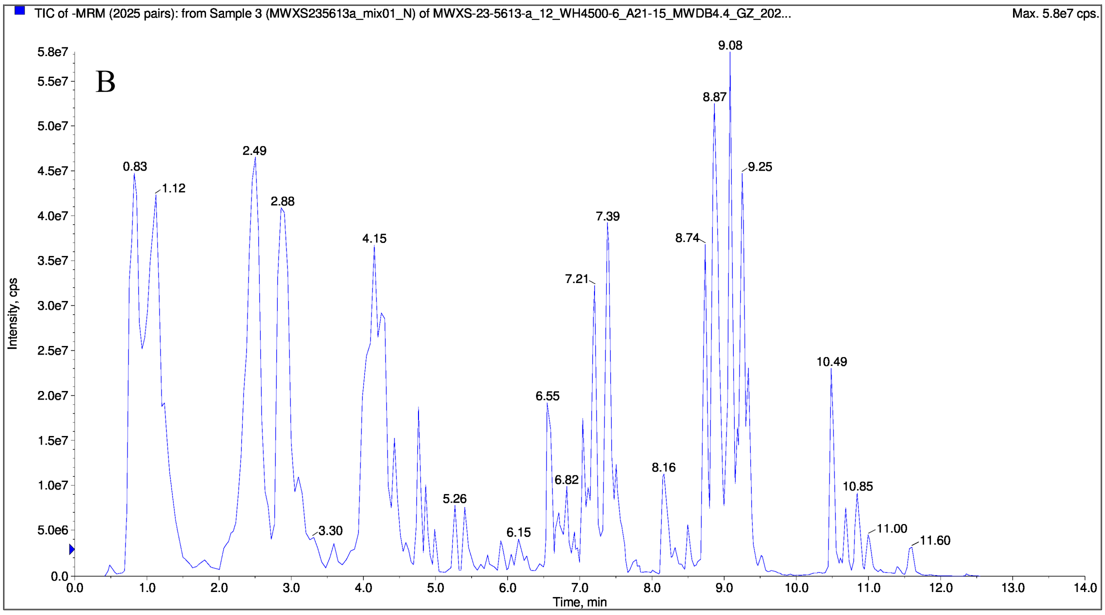

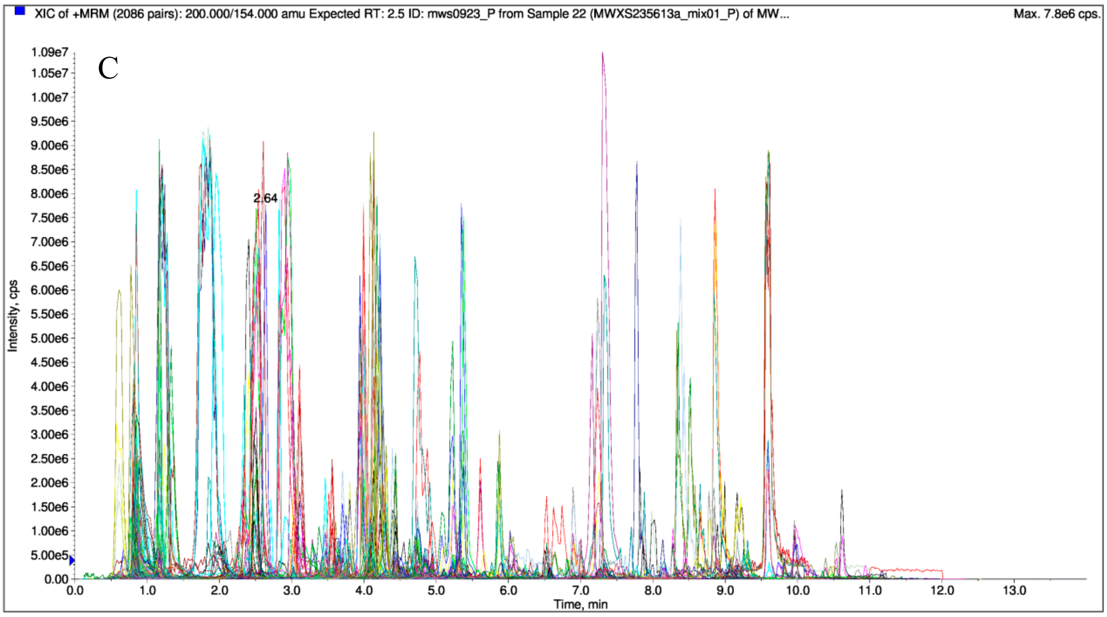

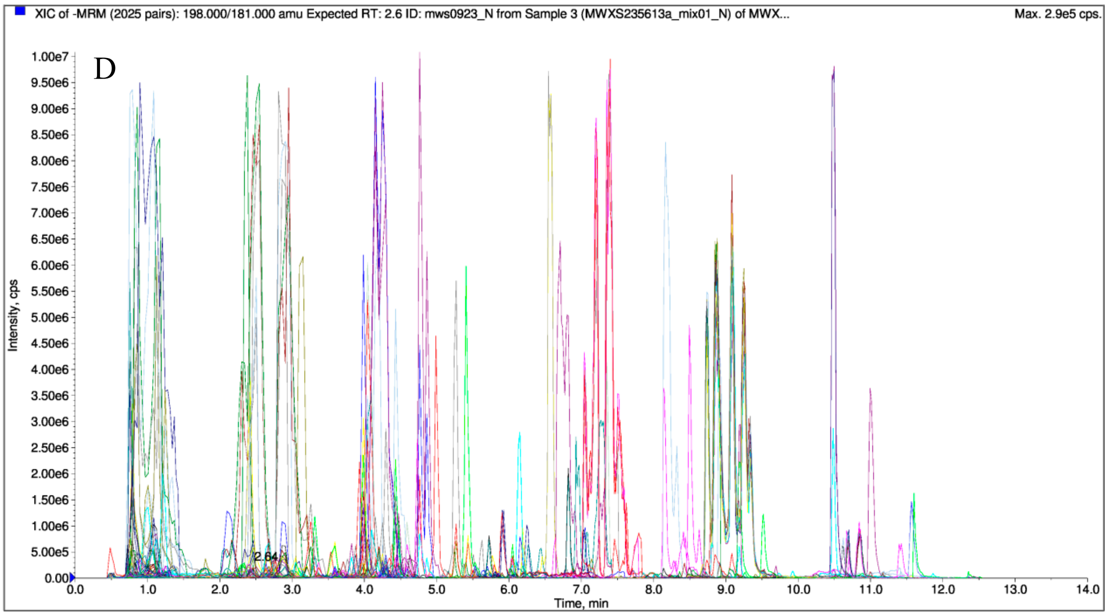

Figure S1. Total ion current diagram of the quality control samples in (A) electrospray ionization (ESI)+ and (B) in ESI– mode. Multi-peak detection plot of the metabolites in multiple reaction monitoring (MRM) in (C) ESI+ mode, and (D) ESI– mode.

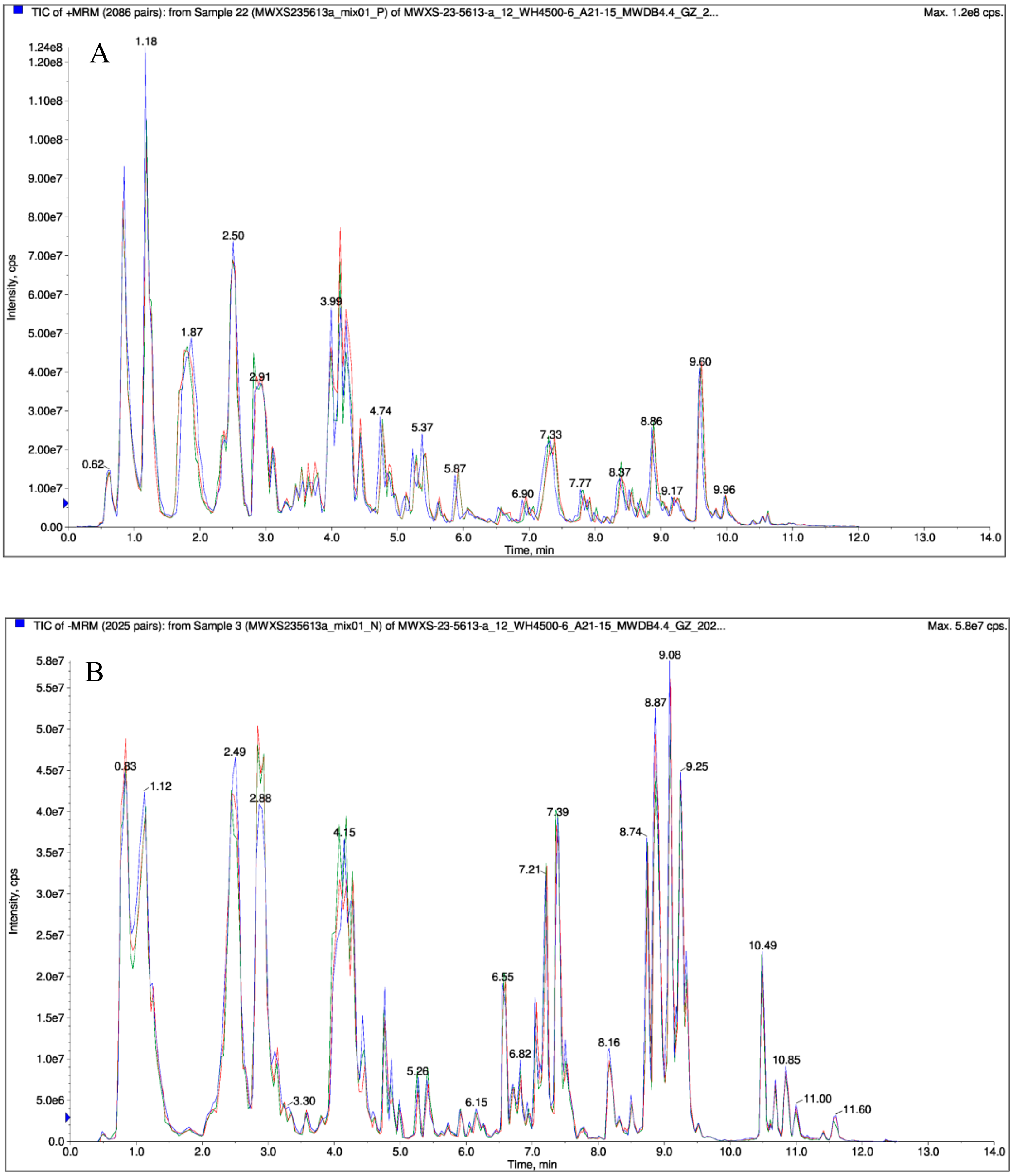

Figure S2. Total ion current overlaps of the three quality control samples using mass spectrometry detection. (A) Total ion current (TIC) overlay plot in electrospray ionization (ESI)+ mode. (B) TIC overlay plot in (ESI) − mode.

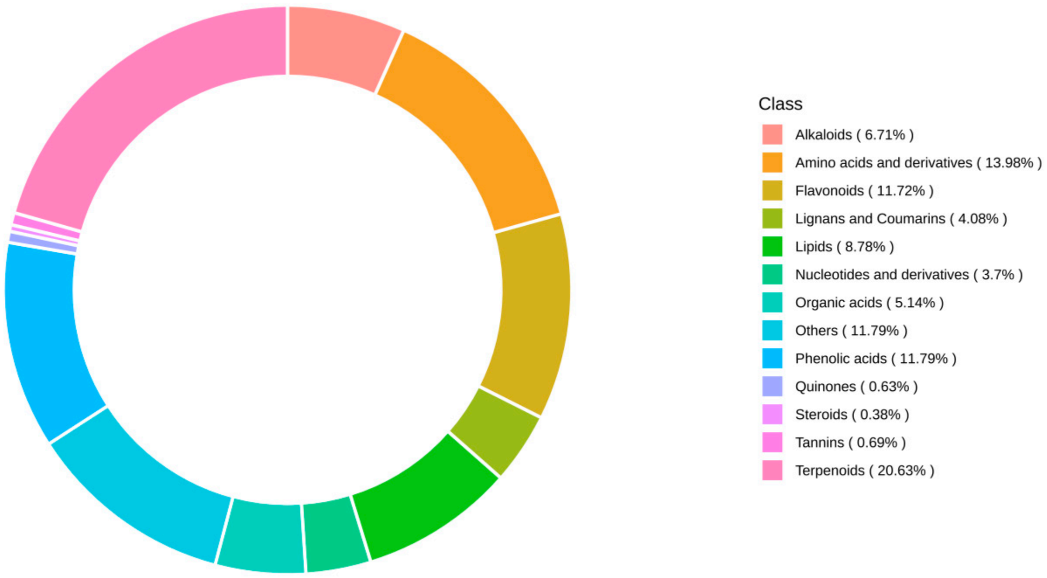

Figure S3. Metabolite classes and quantities detected in the samples from the four growth stages of *A. trifoliata* fruit.

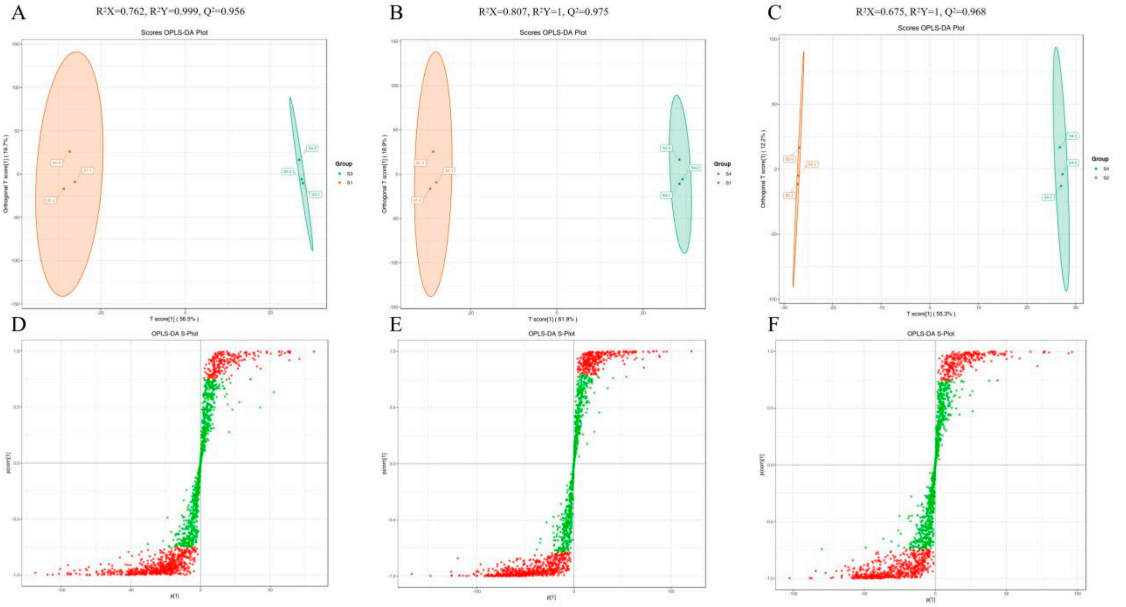

Figure S4. Orthogonal partial least squares discriminant analysis (OPLS-DA) scores. Scores of the OPLS-DA model with (A) S3 vs. S1, (B) S4 vs. S1, and (C) S4 vs. S2. OPLS-DA s-plot model with (D) S3 vs. S1, (E) S4 vs. S1, and (F) S4 vs. S2. Red dots indicate that the VIP values of these metabolites are greater than 1, while green dots indicate that the VIP values of these metabolites are less than or equal to 1.

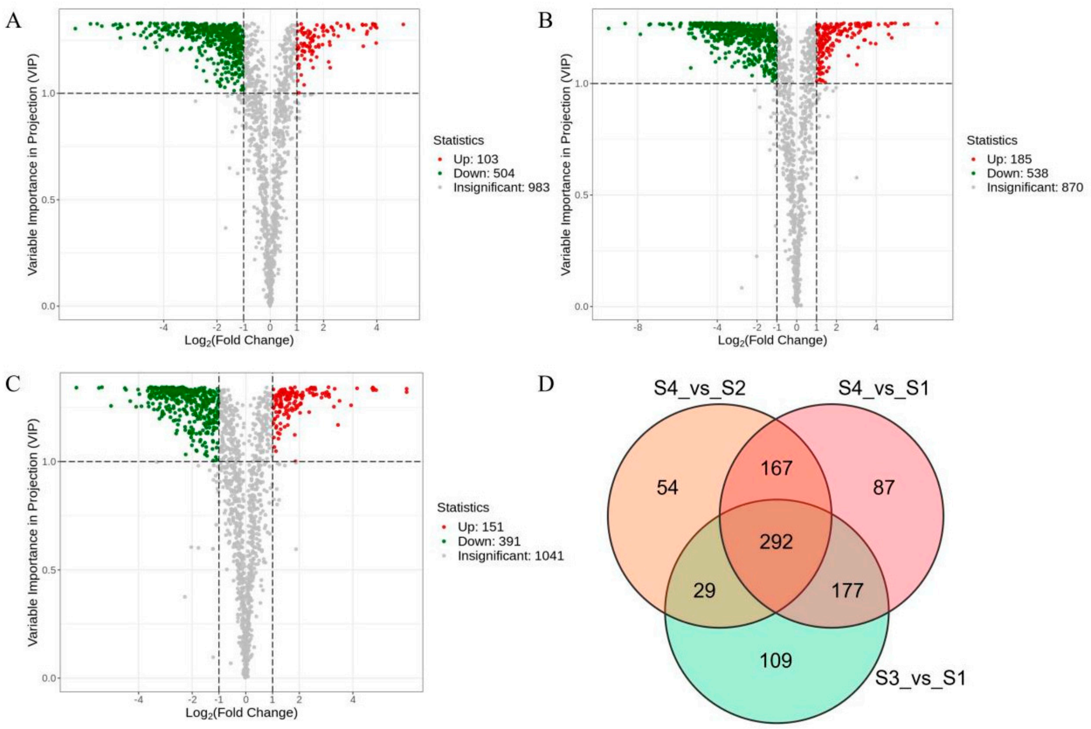

Figure S5. Differential metabolite analysis of *A. trifoliata* fruit at different growth stages. (A–C) Volcano plots of differential metabolites in different pairwise comparisons: (A) S3 vs. S1, (B) S4 vs. S1, and (C) S4 vs. S2. Green dots indicate downregulated, differentially expressed metabolites; red dots indicate upregulated, differentially expressed metabolites; and gray dots indicate detected metabolites with insignificant differences in expression. (D) Venn diagram showing the common and unique metabolites in the comparison groups.
